# Pain intensity estimation based on a spatial transformation and attention CNN

**DOI:** 10.1371/journal.pone.0232412

**Published:** 2020-08-21

**Authors:** Xuwu Xin, Xiaoyan Lin, Shengfu Yang, Xin Zheng

**Affiliations:** 1 The Second Affiliated Hospital of Shantou University Medical College, Shantou, China; 2 The First Affiliated Hospital of Jinan University, Guangzhou, China; 3 Shantou Chaonan Minsheng Hospital, Shantou, China; Politechnika Krakowska im Tadeusza Kosciuszki, POLAND

## Abstract

Models designed to detect abnormalities that reflect disease from facial structures are an emerging area of research for automated facial analysis, which has important potential value in smart healthcare applications. However, most of the proposed models directly analyze the whole face image containing the background information, and rarely consider the effects of the background and different face regions on the analysis results. Therefore, in view of these effects, we propose an end-to-end attention network with spatial transformation to estimate different pain intensities. In the proposed method, the face image is first provided as input to a spatial transformation network for solving the problem of background interference; then, the attention mechanism is used to adaptively adjust the weights of different face regions of the transformed face image; finally, a convolutional neural network (CNN) containing a Softmax function is utilized to classify the pain levels. The extensive experiments and analysis are conducted on the benchmarking and publicly available database, namely the UNBC-McMaster shoulder pain. More specifically, in order to verify the superiority of our proposed method, the comparisons with the basic CNNs and the-state-of-the-arts are performed, respectively. The experiments show that the introduced spatial transformation and attention mechanism in our method can significantly improve the estimation performances and outperform the-state-of-the-arts.

## Introduction

As one of the important indicators of our health, pain is an unpleasant feeling caused by illnesses, injuries or mental distress. In the medical field, pain is often considered as the fifth vital sign [1]. Especially in the case of not optimistic, chronic pain may bring a variety of pathological and physiological risks. However, either in a clinical inspection or using Visual Analog Scale (VAS) [2], the doctor cannot understand the pain of the patient, that is, the feeling of pain is often subjectively stated by the patient. This self-reported pain assessment is very subjective and has certain flaws: (1) the self-reported mechanism is useless for the people who cannot express their pain intensity (e.g., newborns, post-operative patients, etc.) [3, 4]; (2) different individuals always experience the same pain differently, making it difficult for doctors to obtain accurate pain assessments. In addition, studies have shown that human pain is mainly reflected in the changes of our facial expressions, which can provide the most reliable and accurate source of information regarding a subject’s health condition. Therefore, developing a technique that can automatically assess the pain intensity from a patient’s face is essential for telemedicine and for groups that do not effectively express pain perception, as well as for future smart healthcare. For instance, it can monitor the patient’s pain state autonomously, without the need for a caregiver to observe all day, and it can also provide doctors with alerts when severe pain occurs.

**Fig 1:**
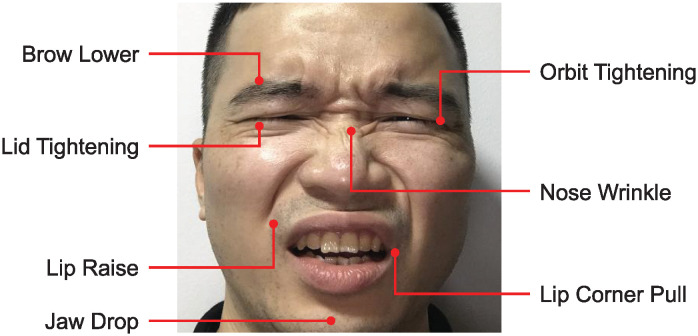
Facial action coding of seven component actions when pain occurs. It is noted that the picture was collected by the author himself and agreed to be published in Plos One.

The current automatic pain assessment techniques mainly solve the problem of pain intensity estimation by analyzing facial expressions. This is because the face is indeed an important source of information about health conditions [5], and facial expressions are thought to be the spontaneous responses to painful experiences in humans. Most researches on facial expression are based on Facial Action Coding System (FACS) [6], which can score facial expressions according to elementary facial Action Units (AUs). Each AU is coded with onset, offset, and an intensity on a five-point scale. Fig 1 illustrates the related coding AUs when facial pain occurs.

Over the past decade, many evaluation methods have been proposed and achieved satisfactory performances [7–12]. However, the interference of background generated during the video capture process and the weight distribution of the face region during evaluation have not been well considered [12]. At present, most methods directly estimate pain intensity based on whole face image with background information, although some works have divided face image into different regions [11, 12]. However, these estimation methods require extensive hand-designed rules. For instance, Huang *at al*. [12] divided the face image equally into four regions, instead of considering the impact of different regions on pain estimation. Therefore, in this paper, we propose a new pain intensity estimation method, which can adaptively assign weights to different regions of the face image and transform the irregular face image to eliminate background interference.

**Fig 2:**
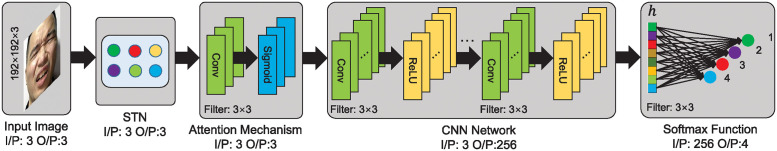
The architecture of the proposed pain intensity estimation method, where I/P and O/P denote the channel number of input and output feature maps, respectively. In the proposed method, the input face image is first fed into STN to against background interference; then, the attention mechanism is used to adaptively distribute different face region weights; finally, the attentional face image is input to CNN module and Softmax Function to extract features and classify pain levels.

Fig 2 shows the architecture of the proposed pain intensity estimation method. The face image is first fed into a spatial transformation network to eliminate the interference of background; then, the attention mechanism is used to weight the transformed face image; after that, a convolutional neural network (CNN) is introduced to extract the self-learned features for describing pain intensity, and a classifier is explored to estimate the pain intensity of the input face images. Among the contributions of this present paper, we can summarize them as follows:

1. While most previous works on pain intensity estimation are based on whole face image, we propose a novel and appealing approach using spatial transformation and attentional information, and demonstrate that the proposed method can be very useful in recognizing different levels of pain intensity.
2. The spatial transformation and attention mechanism are used to address with the problems of background interference and adaptive weight distribution, respectively. Extensive experiments and analysis on the UNBC-McMaster shoulder pain database show that the proposed method can outperform the basic CNN method and the-state-of-the-art methods.

The remainder of the paper is organized as follows: Section of Related Work reviews the existing state-of-the-art methods for pain intensity estimation. In Section of Proposed Method, we describe the proposed spatial transformation and attentional CNN method. Section of Experimental Results and Discussion gives the details of the experimental protocol and reports the obtained results. In Section of Conclusion, we conclude the paper and discuss some directions for future work.

## Related work

In recent years, many methods have been developed to tackle the problem of automatic pain intensity estimation. Depending on the outputs of the algorithms, existing methods can be further divided into two categories: (1) determining the presence of pain and (2) measuring the intensity of pain. For the former methods, they mainly designed models that automatically recognize pain from painlessness [13–15]. For instance, Brahnam *at al*. [16] described pain images by using Discrete Cosine Transform (DCT) and reduced the dimension with Sequential Forward Selection (SFS) algorithm, where the nearest neighbor is used for pain classification. In another work, a correlation vector machine (RVM), a Bayesian extension of the support vector machine (SVM) algorithm, is applied to manually select facial images [17]. Considering the texture differences of different pain levels, Guo *at al*. [7] exploited local binary pattern (LBP) features and its variants to capture texture information of face images. Apart from texture difference, shape is also another important clue for pain detection. Ashraf *at al*. [13] used the active appearance model (AAM) to detected face key points, and analyze the pain face shape in view of the detected key points. By invoking AAM, Luceyetal *at al*. [14] aligned the face images of manually labeled keyframes and feed them into an SVM classifier for frame-level pain recognition.

With regard to the measure of pain intensity, according to the aforementioned pain intensity metric [18], pain expression can be further classified into several discrete levels. Therefore, the most recent works about automatic pain assessment have focused on the challenging task of estimating pain intensity instead of classifying pain or non-pain. More specifically, Lucey *at al*. [4] utilized extended SVM classifiers to estimate pain intensity of three levels. In another work, Kaltwang *at al*. [19] computed LBP and DCT features from facial images and combined them to classify different pain levels, where the relevance vector regression was used for classification task. Hammal and Cohn [15] extracted hand-crafted features based on Log normal filters to identify four pain intensity levels. Florea *at al*. [20] accomplished pain intensity recognition task by using a histogram of topographical features and an SVM classifier, and improved the estimation performance. Recently, Zhao *at al*. [10] took advantage of the natural onset-apex-offset evolution pattern of facial expression to regress intensity estimation.

More recently, with the successes of deep learning in computer vision [21–23], some works have introduced deep neural networks into pain intensity estimation instead of using conventional hand-crafted features. For instance, Huang *at al*. [12] fed the divided face images into four different CNN models and concatenated the fully connected layers to estimate pain intensity. Yang *at al*. [11] combined high-level features of CNN with low-level LBP features of key patches for pain description. Apart from extracting features from one video frame, temporal information within video sequences are also computed by deep neural networks. For instance, Zhou *at al*. [24] converted video frames into vectors and input them into a Recurrent Convolutional Neural Network (RCNN) to regress the pain intensity. In another work, Rodriguez *at al*. [25] first extracted self-learned features of each frame via the fully connected layer of a CNN architecture, then fed the extracted features to a Long-Short Term Memory (LSTM) [26] to obtain the temporal information.

The aforementioned methods based on hand-crafted features or deep learning have achieved satisfactory performances, but the background interference and the adaptive facial region weight distribution that may be encountered in pain estimation have not been well considered. Based on this, we propose an automatic pain estimation algorithm in this paper.

## Proposed method

In order to against the problems of background interference and facial region adaptive distribution weights, we propose a spatial transformation and attention CNN for pain intensity estimation. The overall estimation pipeline is shown in Fig 2, which consists of five modules: Input Image, STN, Attention Mechanism, CNN Network and Softmax Function. More specifically, the input face image is first provided as input to a STN module for address with background interference; then, the attention mechanism is used to distribute different weights of different face regions; after that, the attentional face image is input into the CNN module to extract feature descriptors; finally, the outputs of the CNN module is measured by the Softmax function, which is further used to optimize the parameters of the STN, Attention Mechanism and CNN modules in back propagation process.

### Spatial transformation network

**Fig 3:**
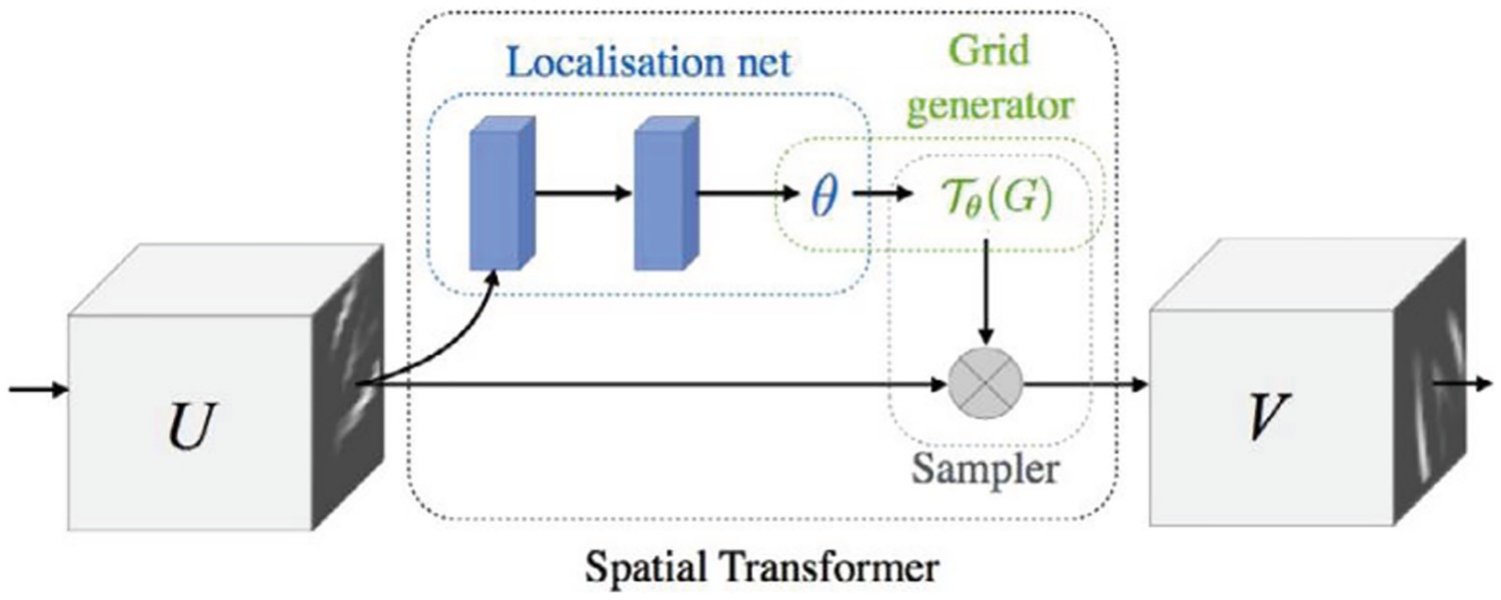
The components of spatial transformer network [27].

For the face image *I*, we normalize it to [0, 1] by the operation *I*/255 denoted as *I*′. Then, the normalized face image *I*′ is fed into the spatial transformation network (STN) [27] to perform a geometric transformation. So that the proposed method is provided with the ability of spatially invariant to the input face image in a computationally efficient manner. As shown in Fig 3, the STN consists of three elements: the localisation network, the grid generator and the sampler.

More specifically, the localisation network *f*_*loc*_ takes an input feature map *U* ∈ *R*^*H*×*W*×*C*^, where *H*, *W* and *C* are the height, width and channels of *U* respectively, and outputs the parameters *θ* of the transformation *T*_*θ*_ to be applied to the feature map *θ* = *f*_*loc*_(*U*). The dimension of *θ* depends on the type of transformation *T*_*θ*_ that will to be parameterized. In STN of the proposed method, *θ* includes 6-dimensional parameters. This is because the *T*_*θ*_ performs a 2D affine transformation, which allows translation, cropping, rotation, scaling, and skewing. Detailed architecture of the localisation network is drawn in Table 1. In transformation process, the 6-dimensional *θ* is used in grid generator to create a sampling grid for obtaining the desired transformed output. Finally, the sampler component is utilized to produce the transformed output feature map *V* by performing a bilinear sampling of the generated sampling grid and the input feature map *U*. Here, *U* is the normalized face image *I*′, and *H*, *W* and *C* equal to 192, 192 and 3 respectively. For each source coordinate ($x_{i}^{s},y_{i}^{s}$) of *I*′ and the transformation matrix *A*_*θ*_, the target coordinates of the regular grid in the output feature map ($x_{i}^{t},y_{i}^{t}$) can be written as Eq 1.

$$\begin{array}{c} \left(\begin{array}{c} x_{i}^{s}\\ y_{i}^{s} \end{array})=A_{\theta }(\begin{array}{c} x_{i}^{t}\\ y_{i}^{t}\\ 1 \end{array})=[\begin{array}{c} \theta_{11}\;\theta_{12}\;\theta_{13}\\ \theta_{21}\;\theta_{22}\;\theta_{23} \end{array}](\begin{array}{c} x_{i}^{t}\\ y_{i}^{t}\\ 1 \end{array}\right) \end{array}$$ (1)

**Table 1:** Localisation network details of spatial transformers used for the normalized face image.

layer	1	2	3	4	5	6	7	8	9	10	11
type	Conv	ReLU	mPool	Conv	ReLU	mPool	Conv	ReLU	mPool	Conv	ReLU
filt size	[3, 3]	−	−	[3, 3]	−	−	[3, 3]	−	−v	[3, 3]	−
filt dim	3	−	−	16	−	−	16	−	−	16	−
num filts	16	−	−	16	−	−	16	−	−	16	−
stride	[2, 2]	1	[2, 2]	[2, 2]	1	[2, 2]	[2, 2]	1	[2, 2]	[2, 2]	1
pad	[1, 1]	0	0	[1, 1]	0	0	[1, 1]	0	0	[1, 1]	0
layer	12	13	14	15	16	17	18	19	20	21	−
type	mPool	Conv	ReLU	mPool	Conv	ReLU	mPool	FC	ReLU	FC	−
filt size	−	[3, 3]	−	−	[3, 3]	−	−	[1, 1]	−	[1, 1]	−
filt dim	−	16	−	−	16	−	−	144	−	32	−
num filts	−	16	−	−	16	−	−	32	−	6	−
stride	[2, 2]	[2, 2]	1	[2, 2]	[2, 2]	1	[2, 2]	[2, 2]	1	[2, 2]	−
pad	0	[1, 1]	0	0	[1, 1]	0	0	0	0	0	−

### Attention mechanism

**Fig 4:**
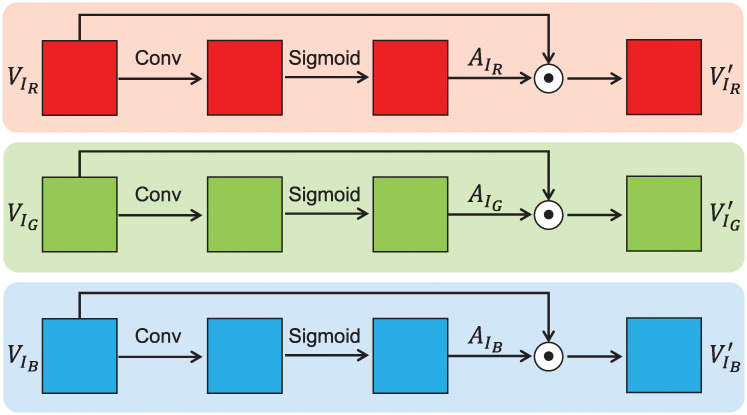
The flow chart of attention mechanism.

In the process of pain intensity estimation, different face regions should have different weights for estimation results [11, 12]. For instance, the action units play a more important role in recognizing pain intensity levels than other regions, as shown in Fig 1. Therefore, after STN, each channel of transformed color face images is fed into the module of attention mechanism to obtain the self-learned weights of different regions. Then, the transformed color face images are multiplied by the self-learned weights for computing attentional face images. The detailed attention mechanism is shown in Fig 4. Specifically, for the transformed *V*_*I*_, each color channel (i.e., $V_{I_{R}}$, $V_{I_{G}}$ and $V_{I_{B}}$) is input into a convolutional (Conv) layer with kernel size of 3×3 and padding size of 1×1. After that, the convolutional feature maps are activated with *Sigmoid* function to compute the attentional weights denoted as $A_{I_{R}}$, $A_{I_{G}}$ and $A_{I_{B}}$ respectively. At end, the computed attentional weights are multiplied by the transformed face images to get the attentional face images. The attention mechanism can be written as Eq 2.

$$\begin{array}{c} V_{I}^{\prime }=V_{I}*Sigmoid\left(Conv\left(V_{I}\right)\right) \end{array}$$ (2)

where $V_{I}^{\prime }$ denotes the attentional face image, * denotes matrix point multiplication, *Conv*(⋅) is the convolution operation, *V*_*I*_ is the output of STN, and *Sigmoid*(*x*) = 1/(1 + *e*^−*x*^).

### CNN network

As shown in Fig 2, the CNN network module consists of *n* serial convolutional and ReLU layers. The purpose of this module is to extract self-learned features from the attentional face image $V_{I}^{\prime }$. More specifically, a convolutional layer with filter size of 3×3×3×64 and a rectified linear unit (ReLU) layer are firstly used to process $V_{I}^{\prime }$. For the size of the convolution filter with dimension of 3×3×3×64, the first two dimensions (3×3) represent the size of the convolution kernel, the third dimension (3) denotes the number of input feature maps, and the last dimension (64) represents the number of output feature maps. With regard to ReLU, it has strong biological and mathematical underpinning [28] and was demonstrated to further improve training of deep neural networks [29]. Compared with other activation functions (such as *Sigmoid*, *Tanh*, etc.), ReLU function has a wider activation area, which can effectively prevent the diffusion of training gradients. After the first ReLU layer, the feature maps are pooled through a maximum pooling layer to reduce the spatial dimension. Repeated five times in this way, the finally feature maps with size 3×3×256 are output by the final pooling layer, and denoted as $f_{V_{I}^{\prime }}=C\left(V_{I}^{\prime },W\right)$, where *C*(⋅) denotes the feature extraction function of the CNN network and *W* represents the parameters of *C*. It is worth mentioning that the filter size of all other convolutional layers is 3×3, allowing for deep models with a low number of parameters [30]. The hierarchic architecture of the CNN network is shown in Table 2.

**Table 2:** The configuration parameters of the CNN network module.

layer	1	2	3	4	5	6	7	8	9
type	Conv	ReLU	mPool	Conv	ReLU	mPool	Conv	ReLU	mPool
filt size	[3, 3]	−	−	[3, 3]	−	−	[3, 3]	−	−
filt dim	3	−	−	64	−	−	64	−	−
num filts	64	−	−	64	−	−	128	−	−
stride	[2, 2]	1	[2, 2]	[2, 2]	1	[2, 2]	[2, 2]	1	[2, 2]
pad	[1, 1]	0	0	[1, 1]	0	0	[1, 1]	0	0
layer	10	11	12	13	14	15	16	17	18
type	Conv	ReLU	mPool	Conv	ReLU	mPool	Conv	ReLU	mPool
filt size	[3, 3]	−	−	[3, 3]	−	−	[3, 3]	−	−
filt dim	128	−	−	128	−	−	256	−	−
num filts	128	−	−	256	−	−	256	−	−
stride	[2, 2]	1	[2, 2]	[2, 2]	1	[2, 2]	[2, 2]	1	[2, 2]
pad	[1, 1]	0	0	[1, 1]	0	0	[1, 1]	0	0

### Softmax function

For pain intensity recognition, its essence is to classify different levels of pain intensity. Therefore, after the CNN network module, a FC layer (denoted as *L*) with four neurons is introduced and the most commonly used *Softmax* loss function is used to measure the estimation error [31]. In network training, the *Softmax* loss function can maximize the probability of the right class and update the network parameters based on the algorithm of back propagation (BP) [32], as illustrated in Eq 3.

$$\begin{array}{c} \begin{array}{c} C=-\sum_{j=1}^{T}\left\{y_{j}log\left(\frac{e^{a_{j}}}{\sum_{j=1}^{T}e^{a_{j}}}\right)\right\} \end{array} \end{array}$$ (3)

where *T* is the levels of pain intensity (here *T* = 4), *y*_*j*_ is *j*_*th*_ value of the one-hot label of training sample, and *a*_*j*_ represents the output of the *j*_*th*_ neuron of *L*. In testing stage, we classify the pain intensity of the input face image *I* based on the probability value of the neuron output of *L*.

### Implementation details

In order to ensure that all convolutional layers have approximately the same output distribution and to improve the rate of convergence, the parameters of the CNN model are initialized using [33], as shown in Eq 4.

$$\begin{array}{c} W_{i}^{l}=\frac{rand(n_{l})}{\sqrt{2/n_{l}}} \end{array}$$ (4)

where $W_{i}^{l}$ is the parameter of *l*_*th*_ convolutional layer of *i*_*th*_ CNN, *rand*(⋅) samples from a zero mean, unit standard derivation gaussian function, and *n*_*l*_ is the channel number of inputs in convolutional layer. In training stage, the momentum *β* of SGD is set to 0.9, the learning rate *α* is set to 10^−4^ and all mini-batches are traversed and re-allocated randomly. All face images are normalized into 192×192×3 with the scale ranging from 0 to 1. The proposed pain intensity estimation network is implemented using the toolbox of PyTorch with the version 1.0.0.

## Experimental results and discussion

In this section, we report and discuss the pain estimation results achieved by the proposed spatial transformation and attention CNN. Firstly, the database used to validate our method are introduced. Secondly, we evaluate the effectiveness of the used spatial transformation network and attention mechanism. Finally, the proposed method is compared with the-state-of-the-art methods.

### Experimental data

In order to validate the effectiveness of our proposed method, we test it on the publicly available UNBC-McMaster Shoulder Pain Expression Archive Database [34]. The database contains in total 200 video sequences of FACS coded frames from 25 subjects. The subjects are of various occupations and age groups. These subjects are self-identified as suffering from shoulder pain and the videos are recorded when they are experiencing a series of active and passive motions of their affected and unaffected limbs. In this database, each frame is AU-coded by certified FACS coders, where there are 44 individual action units (AUs) in FACS. The corresponding prkachin and solomon intensity (PSPI) scores [18] are computed in 16 discrete levels (0-15) to estimate different pain intensities. For the used PSPI scores, they are calculated based on six specific action units of FACS. More specifically, this is because Prkachin *at al*. [35] found that four facial actions—brow lowering (AU4), orbital tightening (AU6 and AU7), levator contraction (AU9 and AU10) and eye closure (AU43)—carried the bulk of information about pain, where *AU*4, *AU*6, *AU*7, *AU*9, *AU*10 and *AU*43 are the Action Units. The calculation of PSPI is accorded to Eq 5:

$$\begin{array}{c} Pain=AU4+max(AU6,AU7)+max(AU9,AU10)+AU43 \end{array}$$ (5)

where *max*(⋅) is the operation of selecting the maximum value.

In this paper, as in [10, 12, 15], we integrate the pain into four different levels corresponding to PSPI. More specifically, the scores of 0 are integrated into the first pain level, the scores ranging from 1 to 2 are integrated into the second pain level, the scores ranging from 3 to 5 are integrated into the third pain level, and other scores are integrated into the fourth pain level. With regard to the four pain levels, the corresponding pain states are no pain, weak pain, intense pain and excruciating pain, respectively. The more detailed sample distribution of different pain levels is shown in Table 3. Considering the training efficiency, the database is randomly divided into three disjoint subsets (i.e., training set, development set and test set), where the training set contains 10 subjects, the validation set contains 5 subjects, and the test set contains 10 subjects. During the experiment, the training set is used to update the network parameters, the development set is used to select the best network, and the test set is used to evaluate the network performance. It is noted that before dividing subsets, we randomly select 5260 samples from pain level 0 to solve imbalance data problem. With regard to the performance evaluation, the results are reported in term of classification accuracy, precision, recall and mean squared error (MSE) [19]. This study used the UNBC-McMaster Shoulder Pain Expression Archive Database and all data were fully anonymized before the authors accessed them.

**Table 3:** The detailed sample distribution and number of different pain levels.

Pain Intensity	PSPI	Description	Amount	Portion
Level 0	0	None	40007	82.7%
Level 1	1-2	Weak	5260	10.9%
Level 2	3-5	Intense	2456	5.1%
Level 3	6-16	Excruciating	653	1.3%

### Analysis of STN and attention mechanism

In order to solve the problem of background interference and adaptive weight distribution, we propose a pain intensity estimation method based on a spatial transformation and attention CNN. Therefore, in this part, the effectiveness of the STN and attention mechanism are analyzed respectively. More specifically, the method with STN and with attention mechanism, the method with no STN and attention mechanism, the method with STN and with no attention mechanism and the method with no STN and with no attention mechanism are compared with each other. The comparison results and the confusion matrixes of different modes are shown in Tables 4 and 5, respectively.

**Table 4:** The precision (%), recall (%), accuracy (%) and MSE of different modes of the proposed method. P denotes to precision, R denotes recall, A denotes classification accuracy, and Att is short for attention.

Mode	Level 0		Level 1		Level 2		Level 3		A	MSE
	P	R	P	R	P	R	P	R		
No-STN-No-Att	42.6	58.9	11.4	10	13.7	5.8	0	0	32.7	2.5358
STN-No-Att	53.4	89	32.6	13.3	34.4	8.6	0	0	48.8	1.4033
No-STN-Att	52.6	88	33	14.7	16.1	3	0	0	47.8	1.3185
STN-Att	54.5	91.3	40.5	16	42.2	10.5	0	0	51.1	1.1014

**Table 5:** Confusion matrixes of different modes of the proposed method.

No-STN-No-Attention	Level 0	Level 1	Level 2	Level 3
Level 0	2254	1443	312	118
Level 1	1946	231	80	43
Level 2	916	263	78	89
Level 3	175	96	98	0
STN-No-Attention	Level 0	Level 1	Level 2	Level 3
Level 0	3406	292	59	70
Level 1	1891	305	66	38
Level 2	890	253	116	87
Level 3	187	86	96	0
No-STN-Attention	Level 0	Level 1	Level 2	Level 3
Level 0	3368	297	64	98
Level 1	1882	337	51	30
Level 2	974	295	40	37
Level 3	183	92	94	0
STN-Attention	Level 0	Level 1	Level 2	Level 3
Level 0	3495	246	42	44
Level 1	1870	368	47	15
Level 2	877	199	141	129
Level 3	170	95	104	0

For the STN, we can clearly find that compared to the basic CNN network, the CNN with STN can improve the classification accuracy from 32.68% to 48.80% and reduce the MSE from 2.5358 to 1.4033. With regard to the recall and precision measures, compared with other modes, the proposed method with STN and attention mechanism achieved the best results. For instance, when the pain level is 0, the recall rate of Level 0 is 91.3%, and the precision of Level 0 is 54.4%. However, for samples with Level 3, its recall and precision are both 0%, which means that all samples with Level 3 are misclassified as other pain levels. As shown in Table 5, the proposed method with STN and attention mechanism classified 369 samples with Level 3 as Level 0, Level 1 and Level 2 with a number of 170, 95 and 104, respectively. By analyzing samples of different pain levels, there are some training samples that are difficult to classify. We speculate that the confusing and imbalanced training data makes the algorithm’s unsatisfactory on Level 3.

For the inputs of STN module, there are some background information, and these backgrounds have no effect on pain estimation. Therefore, after training, the STN module performs 2D affine transformation on the original image to eliminate the background interference. Regarding the reduced background interference, since the input of the STN module is a face region containing a small amount of background, the reduced background noise is not significant compared with the input face images. However, it can be found that the output of the STN is to reduce as much surrounding background noise as possible. With regard to the additional black edges in the transformed images, we analyze that this is caused by the operation of image rotation. In the process of feature extraction and classification, these black edges only occur in a limited edge area and the pixel values equal to 0, which has little effect on the final classification result.

For the introduced attention mechanism, the CNN with attention mechanism can improve the classification accuracy from 32.68% to 47.76% and reduce the MSE from 2.5358 to 1.3185. From the attentional regions, it can conclude that the attention mechanism can adaptively assign different weights to different facial regions. More specifically, the key AUs around the eyes and cheeks have higher weights.

**Fig 5:**
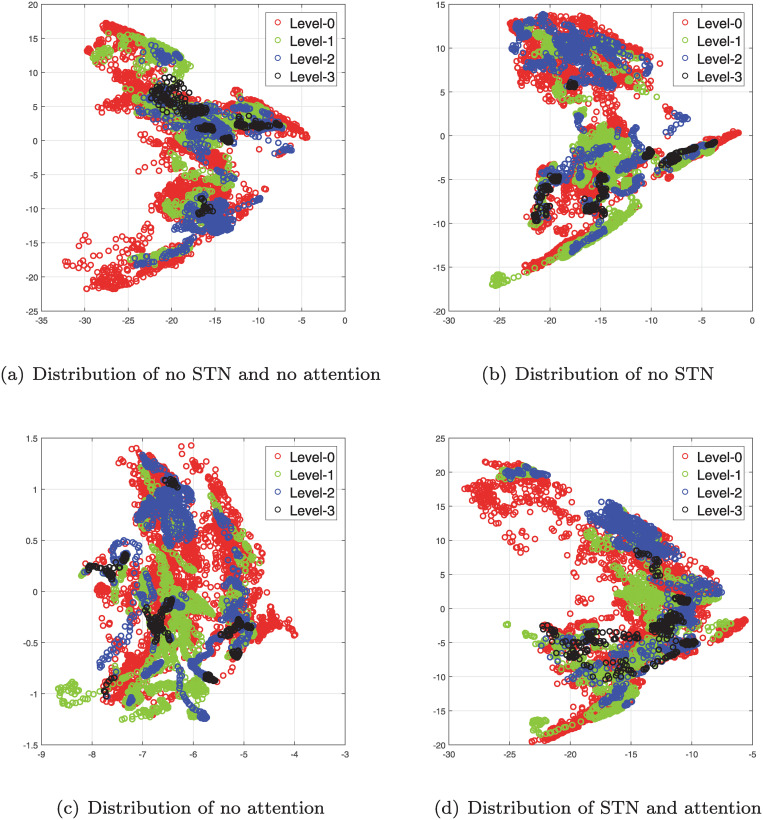
The feature distribution in different modes. First, the PCA algorithm is used to reduce the feature (i.e., the response of the last fully connected layer) dimension to 2. Then, we plot the reduced features in a 2D space.

Therefore, considering the effectiveness of STN and attention mechanism, we combine them in the proposed method. From Table 4, it can be found that the proposed method with STN and attention mechanism can obtain the best estimation results, that is, *Accuracy* = 51.06% and *MSE* = 1.1014. Compared the method only with STN or attention mechanism, the method combined both of them effectively solves the problems of background interference and weight distribution. Fig 5 visualizes the sample distribution of the UNBC-McMaster database in different combination modes. As the presented sample distributions, even under our proposed method, the pain samples of different levels are not completely separated, which also illustrates the difficulty of the pain estimation problem with weak texture differences. However, compared with the basic method with no STN and no attention mechanism, the samples are more distinguishable under our proposed feature space.

### Comparison with the-state-of-the-art methods

In the paper, we test the state-of-the-art pain estimation approaches and our proposed method using same samples. Table 6 shows the comparison results, and Table 7 presents more detailed distribution of classification results. It can be seen that the classification accuracy of our proposed method is 51.06%, and the MSE is 1.1014. Compared with other methods, especially with the CNN based method [12] that divides face image into different regions, the classification accuracy is improved about 44%, and the MSE is reduced about 51%, respectively. For the recall and precision measures of each pain level, our proposed also achieved best performance. For instance, the precision of Level 0 has been improved from 34.7% to 54.5%. With regard to Level 3, due to the confusing and unbalanced training samples, both recall and precision measures are unsatisfactory.

**Table 6:** Comparison our proposed method with the state-of-the-art methods. P denotes to precision, R denotes recall, A denotes classification accuracy, and Att is short for attention.

Method	Level 0		Level 1		Level 2		Level 3		A	MSE
	P	R	P	R	P	R	P	R		
Head Analysis [36]	38.9	48.7	12.2	11.5	10.2	4.9	0	0	28.1	3.0127
LBP [37]	34.7	41.9	7.5	7.3	8	3.9	0	0	23.3	3.3342
LPQ [37]	34.7	42	7.7	7.5	8.2	3.9	0	0	23.4	3.3305
BSIF [37]	34.7	42.3	8.2	8	9	4.3	0	0	23.7	3.2825
CNN [12]	44.5	61.4	15.6	12.1	24.4	10.2	0	0	35.3	2.2683
Our Method	54.5	91.3	40.5	16	42.2	10.5	0	0	**51.1**	**1.1014**

**Table 7:** Confusion matrixes of the state-of-the-art methods.

Head Analysis [36]	Level 0	Level 1	Level 2	Level 3
Level 0	1870	1457	415	85
Level 1	1934	264	72	30
Level 2	831	348	66	101
Level 3	172	103	94	0
LBP [37]	Level 0	Level 1	Level 2	Level 3
Level 0	1604	1650	423	150
Level 1	2001	167	84	48
Level 2	844	325	52	125
Level 3	178	97	94	0
LPQ [37]	Level 0	Level 1	Level 2	Level 3
Level 0	1606	1669	417	135
Level 1	1997	173	84	46
Level 2	846	323	53	124
Level 3	180	96	93	0
BSIF [37]	Level 0	Level 1	Level 2	Level 3
Level 0	1618	1659	419	131
Level 1	1995	184	77	44
Level 2	846	319	58	123
Level 3	183	92	94	0
CNN [12]	Level 0	Level 1	Level 2	Level 3
Level 0	2349	1107	252	119
Level 1	1915	278	65	42
Level 2	839	305	137	65
Level 3	173	89	107	0

From the comparison results, it can be found that the background information is an interference that should be considered for identifying different pain levels. In addition, the attention mechanism with the ability to adaptively assign weights can effectively improve the performance of the algorithm. However, in terms of the measures of recall, precision, accuracy and MSE, the performance of our method is still unsatisfactory. More specifically, the recall rates of different pain levels are 91.3%, 16%, 10.5% and 0%, respectively. As aforementioned, this is caused by confusing and unbalanced training samples. Therefore, studying how to eliminate the problem of imbalanced samples and build a more accurate database is the focus of our future research.

## Conclusion

Considering the interference of background and the influence of different facial regions, a spatial transformation and attention CNN is proposed to estimate pain intensity. In the proposed method, the face image is first performed a 2D affine transformation (i.e., translation, cropping, rotation, scaling, and skewing) to against background interference. Then, the transformed result is multiplied with attention weights to balance different facial regions. Extensive experiments on the challenging UNBC-McMaster Shoulder Pain Expression Archive Database showed that our proposed spatial transformation and attentional CNN can effectively improve the estimation performance. However, our proposed method just analyzes still face images and does not effectively use the facial motion information to further improve the pain estimation accuracy. Furthermore, according to the analysis of different pain levels, it is found that the confusing training data is a problem that should be well considered. Therefore, in our future work, we intend to estimate pain intensity from three directions: (1) Based on the existing method, extending a new pain estimation algorithm that can effectively use facial motion information; (2) Establishing or generating a balanced and accurate pain estimation database; (3) Developing a new training mechanism so that the unbalance samples can effectively train network parameters.
